# Sexual Function and Quality of Life in a Cohort of Brazilian Users of Two Kind of Intrauterine Contraceptives

**DOI:** 10.1055/s-0039-1683370

**Published:** 2019-03-25

**Authors:** Jéssica Mayra Ferreira, Andréa Vieira Carreiro, Arlete Fernandes, Luis Bahamondes

**Affiliations:** 1 Department of Gynecology and Obstetrics, Universidade Estadual de Campinas, Campinas, SP, Brazil

**Keywords:** contraception, quality of life, sexuality, contracepção, qualidade de vida, sexualidade

## Abstract

**Objective** To compare sexual function and quality of life (QOL) among intrauterine contraceptive (copper-intrauterine device [Cu-IUD] or the 52-mg 20 µg/day levonorgestrel-releasing intrauterine system [LNG-IUS]) users.

**Methods** This was part of a cross-sectional study. Women aged between 18 and 49 years old, in a heterosexual relationship, reporting sexual intercourse in the previous 4 weeks, using Cu-IUD (Group 1) or LNG-IUS (Group 2) responded to a questionnaire with sociodemographic information, to the Female Sexual Function Index (FSFI), to the World Health Organization QOL Questionnaire Abbreviated Version (WHOQOL-BREF), and to a questionnaire about the contraceptive method used. The Student *t-*test, the Pearson χ^2^ test or the Fisher exact test, and the Mann-Whitney test were used for the analysis. For the adjusted comparison, we have used the analysis of covariance (ANCOVA). A multiple regression analyzing factors related to FSFI ≤ 26.55 was done. Significance was established at *p *< 0.05.

**Results** A total of 347 women in Group 1 (mean age of 32.3 ± 7.5 years old) and of 298 in Group 2 (mean age of 32.7 ± 6.4 years old) completed the questionnaires. Most women had ≥ 8 years of schooling, were in a monogamous relationship, and had had ≤ 2 pregnancies. A total of 122 Cu-IUD and of 87 LNG-IUS users scored ≤ 26.55 on the FSFI. Significant lower scores in physical, environmental, and overall QOL domains in the WHOQOL-BREF questionnaire were found in Group 1. More women using the Cu-IUD were not satisfied with the method.

**Conclusion** We did not find significant differences in sexual function; there was a lower score in some domains of QOL among women who used the Cu-IUD. It was not possible to ensure that those differences were related to the contraceptive method.

## Abstract (pt)

**Resumo:**

**Objetivo** Comparar a função sexual e a qualidade de vida (QV) entre usuárias de contraceptivos intrauterinos (dispositivo intrauterino com cobre [DIU-cobre] ou sistema intrauterino liberador de levonorgestrel [SIU-LNG 52 mg 20 µg/dia].

**Métodos** O presente estudo foi parte de um estudo de corte transversal. Mulheres com idades entre 18 e 49 anos, em relacionamento heterossexual, relatando relação sexual nas 4 semanas anteriores, utilizando DIU-cobre (Grupo 1) ou SIU-LNG (Grupo 2) responderam ao questionário com informações sociodemográficas, ao Índice de Função Sexual Feminino (IFSF), ao Questionário de QV da Organização Mundial de Saúde, versão abreviada (WHOQOL-BREF, na sigla em inglês), e a um questionário sobre o método contraceptivo utilizado criado para o presente estudo. Os testes *t* de Student, o teste χ^2^ de Pearson, o teste exato de Fisher e o teste de Mann-Whitney foram utilizados. A análise de covariância (ANCOVA) foi utilizada para a comparação ajustada. Foi realizada a análise de regressão logística multivariada analisando fatores associados ao IFSF ≤ 26.55. O nível de significância foi *p* < 0.05.

**Resultados** Um total de 347 mulheres no Grupo 1 (idade média 32,3 ± 7,5 anos) e de 298 no Grupo 2 (idade média 32,7 ± 6,4 anos) completaram os questionários. A maioria das mulheres relatou ≥ 8 anos de escolaridade, estava em relação monogâmica e teve ≤ 2 gestações. Um total de 122 e de 87 usuárias do DIU-cobre e do SIU-LNG, respectivamente, pontuaram ≤ 26,55 no IFSF. Pontuações significativamente menores nos domínios físico, meio-ambiente e QV geral no WHOQOL-BREF foram encontrados no Grupo 1. Mais mulheres do Grupo 1 estavam insatisfeitas com o método.

**Conclusão** Não encontramos diferenças na função sexual, entretanto havia menores pontuações em certos domínios da QV entre as usuárias do DIU-cobre. Não foi possível afirmar relação dessas diferenças com os métodos.

## Introduction

Satisfactory sexual life is one of the main components of health and quality of life (QOL).^1^ Female Sexual Dysfunction (FSD) is defined as lack of at least one component in the sexual response cycle (desire, arousal, ability of achieving orgasm, and pain during intercourse).^2^ Furthermore, it has been reported that > 40% of the women complain of 1 or more sexual problems.^1^ ^2^ The possible impacts caused by a contraceptive method in the QOL and sexual function of women may influence their choice for a method or be the cause for its premature discontinuation.^3^ ^4^

Long-acting reversible contraceptive (LARC) methods (intrauterine contraceptives [IUCs], including copper intrauterine device [Cu-IUD] and levonorgestrel-releasing intrauterine system [LNG-IUS], and subdermal implants) are highly safe, cost-effective, suitable for all reproductive ages, and well accepted,^1^ ^5^ especially because they do not require the constant attention of the user.^6^

Despite several advantages, LARC methods may present some side-effects that might affect the general health of women, such as their QOL and sexual health. The Cu-IUD can cause changes in the menstrual pattern, such as spotting or heavier or long menstrual bleeding, and can also cause lower abdominal cramps.^1^ ^7^ The LNG-IUS induces, in many cases, amenorrhea, as well as infrequent or light bleeding, which may be viewed as a benefit by the users because of its convenience; however, this has been reported as one of the reasons for discontinuation, since many women prefer methods that do not change their menstrual pattern.^7^ ^8^

Due to the fact that, to the best of our knowledge, there are no studies that have evaluated both sexual function and QOL among Cu-IUD and LNG-IUS users^1^ ^9^ and that the Cu-IUD and the LNG-IUS induce different bleeding patterns that can influence the QOL and sexual function of their users, we have decided to assess and compare the sexual function and the QOL among users of the Optima copper T intrauterine device (TCu380A IUD) (Injeflex, São Paulo, Brazil) and the LNG-IUS (Bayer Oy, Turku, Finland).

## Methods

This was the data analysis of part of a larger cross-sectional study conducted at the Family Planning Clinic of the Department of Obstetrics and Gynecology of the Faculdade de Medicina of the Universidade de Campinas (UNICAMP), Campinas, state of São Paulo, Brazil. The present study had the approval of the Ethical Committee and informed consent was obtained from all of the individual participants included in the study.

Women attending the Family Planning Clinic for routine care between 1 February 2011 and 31 May 2012 were included if: they were aged between 18 and 49 years old; in use of the Optima TCu380A IUD or of the Mirena LNG-IUS (20µg/day initial release); reported being in a heterosexual relationship for at least 6 months; had engaged in sexual intercourse in the last 4 weeks prior to the study. Those who informed that they had a gynecological disease, who had a known psychiatric disease, including those who were in use of a psychiatric medication, who had any chronic disease, or had a partner with any sexual dysfunction, such as premature ejaculation or impotence, were excluded.

The participants filled out a questionnaire containing sociodemographic information, the Female Sexual Function Index (FSFI), which corresponds to the gold standard method to determine female sexual functioning,^2^ the World Health Organization Quality of Life Questionnaire, Abbreviated Version (WHOQOL-BREF), and a questionnaire formulated for the present study regarding the method in use. In this last multiple-choice questionnaire, the women answered if they had any concerns about getting pregnant while using their chosen method, if they were concerned that the method could cause any damage to their health, if they found the method easy to be used, and if they were satisfied with the method.

The FSFI includes 19 questions divided into 6 domains: desire (2 questions with a score ranging from 1 to 5 points each), arousal (4 questions with a score ranging from 0 to 5), lubrication (4 questions with a score ranging from 1 to 5), orgasm (3 questions with a score ranging from 0 to 5), satisfaction (3 questions with scores ranging from 1 question with scores ranging from 0 to 5 and 2 questions with scores from 1 to 5) and pain (3 questions with a score ranging from 0 to 5). The participants should answer the FSFI questionnaire according to their sexual experience from the previous 4 weeks. Domain scores are derived by summing scores within the domain and multiplying the sum by a factor weight (each domain has a specific factor). A total punctuation was obtained by summing the results from all of the domains. A FSFI total score < 26.55 has been described as the cutoff level for screening for risk of sexual dysfunction.^10^ For the present study, full-scale scores were used to compare the groups of participants.

The WHOQOL-BREF was developed to provide a brief and convenient mean of assessing data for QOL studies. The questionnaire consists of 26 questions with 4 domains (physical health, psychological, social relationship, and environmental) and includes an overall score. The higher the score, the better the QOL. Both the FSFI and the WHOQOL-BREF questionnaires have been validated for the Brazilian population.^11^

### Analysis of the Data

For the data analysis, the use of the contraceptive method was considered as continuous if the IUC user replaced the device for another one on the same day in which the last one was removed at the end of its contraceptive lifespan. The participants were divided into two groups: women who were using the TCu380A IUD and those who were using the LNG-IUS at the time they responded to the questionnaires. The sociodemographic characteristics of the participants are presented as the mean ± standard deviation (SD), or as percentage distributions for categorical variables. The groups were compared regarding the FSFI, the WHOQOL-BREF, and the questionnaire about satisfaction with the method. For these comparisons, we have used the Student *t-*test, the Pearson χ^2^ test, or the Fisher exact test for expected values < 5, and the Mann-Whitney test. For the adjusted comparison, we have used the covariance analysis (ANCOVA). We have also performed a multiple regression with FSFI ≤ 26.55. Significance was established at *p *< 0.05. The Statistical Analysis System (SAS System) for Windows, version 9.2, SAS Institute Inc. 2002–2008, Cary, NC, USA, was used in the analysis.

## Results

From the total of 372 Cu-IUD users (Group 1) and of 313 LNG-IUS users (Group 2) invited to participate in the present study, we have included 347 and 298 from each group, respectively. The other participants were excluded for lack of completeness of the questionnaires. Participants in Group 1 were using the method for 6.0 ± 5.2 months, and in Group 2 for 3.7 ± 5.2 months (*p *< 0.001).

The mean ± SD age of the participants was 32.3 ± 7.5 years old and 32.7 ± 6.4 years old in the Cu-IUD and in the LNG-IUS groups, respectively. More women in Group 1 were non-white, had ≤ 8 years of schooling, and were classified as belonging to the groups C, D or E according to the household income classification (Table 1).

**Table 1:** Sociodemographic characteristics of the participants

Characteristics	TCu380A IUD (*n* = 347)	LNG-IUS (*n* = 298)	*p*-value
*Age (years)*	Mean ± SD (range)	Mean ± SD (range)	
	32.3 ± 7.5 (18–49)	32.7 ± 6.4 (25–47)	0.397*
*Ethnicity*	*n* (%)	*n* (%)	
White	159 (45.8)	198 (66.4)	< 0.001
Non-white	180 (51.9)	92 (30.9)	
Missing data	8 (2.3)	8 (2.7)	
*Schooling (years)*			
Illiterate ≤ 8	0 101 (29.2)	0 37 (12.3)	< 0.001
≥ 8	241 (69.4)	256 (85.7)	
Missing data	5 (1.4)	5 (2.0)	
*BMI*			
< 25.0	154 (44.4)	151 (50.7)	0.228
≥ 25.0	170 (49.0)	126 (42.3)	
Missing data	23 (6.6)	21 (7.0)	
*Marital Status*			
Living with partner	284 (81.8)	239 (80.2)	0.540
Single	62 (17.9)	59 (19.8)	
Missing data	1 (0.3)	0	
*Parity*			
0-2	267 (77.0)	250 (83.9)	0.094^**^
3-4	65 (18.7)	38 (12.7)	
≥ 5	3 (0.8)	38 (12.7)	
Missing data	12 (3.5)	8 (2.7)	
*Vaginal Parity*			
0–2	292 (84.2)	270 (90.6)	0.009^**^
3–4	32 (9.2)	12 (4.0)	
≥ 5	1 (0.3)	0	
Missing data	22 (6.3)	16 (5.4)	
*Employment*			
Paid employment	166 (47.8)	141 (47.3)	0.600
Unemployed, housewife or	179 (51.6)	153 (51.3)	
Missing data	2 (0.6)	4 (1.4)	
*Household Income Classification*†			
A and B	102 (29.4)	150 (50.3)	< 0.001
C, D and E	242 (69.7)	147 (49.4)	
Missing data	3 (0.9)	1 (0.3)	

Abbreviations: LNG-IUS, levonorgestrel-releasing intrauterine system; SD, standard deviation; TCu380A IUD, T-shaped copper intrauterine device.

*Student *t*-test; ** Fisher exact test; other variables: Pearson χ^2^ test. †Household income was grouped based according to the Brazilian Market Research Association's social-class definition based on monthly household income: A: US$5148–US$3720; B: US$2132–US$1191; C: US$655–US$432; D: US$305–US$187; E: ≤ US$186.

Regarding the FSFI, 122 (35.2%) and 87 (25.0%) women presented total a score of ≤ 26.55 in groups 1 and 2, respectively (adjusted *p-value = *0.096). The mean scores of the questionnaire were also analyzed. In both groups, the mean scores were above the cutoff score for sexual dysfunction; however, there were no statistical differences (27.8 ± 4.6 in Group 1, and 29.8 ± 8.0 in Group 2; *p* = 0.796). In the WHOQOL-BREF questionnaire, the mean scores for the physical health, environmental and overall QOL domains were significantly lower in the Cu-IUD group when compared with the LNG-IUS group (Table 2).

**Table 2:** Prevalence of at-risk sexual dysfunction female sexual function index scores and mean quality of life domain scores

Questionnaire	TCu380A IUD (*n* = 347)	LNG-IUS (*n* = 298)	*p*-value*	Adjusted *p*-value**
*Sexual dysfunction FSFI*				
FSFI full-scale score ≤26.55	122 (35.2)	87 (25.0)	0.107	0.096
*WHOQOL-BREF domain scores*				
Physical health	59.9 ± 12.6	78.0 ± 14.3	< 0.001	< 0.001
Psychological	64.7 ± 11.4	65.4 ± 10.0	0.511	0.793
Social relationships	74.5 ± 18.1	74.9 ± 15.3	0.779	0.705
Environmental	59.2 ± 13.4	65.6 ± 12.9	< 0.001	0.004
Overall quality of life	64.5 ± 10.9	71.0 ± 10.3	< 0.001	< 0.001

Abbreviations: FSFI, Female Sexual Function Index; IUD, intrauterine device; LNG-IUS, levonorgestrel-releasing intrauterine system; SD, standard deviation; TCu380A IUD, T-shaped copper intrauterine device; WHOQOL-BREF, World Health Organization quality of life questionnaire abbreviated version.

*Mann-Whitney test; **Analysis of covariance with adjusted values for ethnicity, schooling, vaginal parity, and household income classification.

A total of 227 (65.4%) Cu-IUD users and of 224 (75.2%) LNG-IUS users were satisfied with the method in use, while 56 (16.1%) and 24 (8.0%) were not sure about it in groups 1 and 2, respectively (*p* = 0.012). The other variable about the contraceptive method in use did not differ between the two groups (Table 3).

**Table 3:** Questionnaire with some aspects about the contraceptive method in use

Questions	TCu380A IUD (*n* = 347)	LNG-IUS (*n* = 298)	*p*-value*
*Concerned with getting pregnant while using the method?*			
Very	78 (22.5)	49 (16.4)	0.167
A little	166 (47.8)	144 (48.3)	
Not at all	98 (28.2)	102 (34.2)	
Missing data	5 (1.5)	3 (1.1)	
*Concerned that the method can damage your health?*			
Always	17 (4.8)	15 (5.0)	0.792
Sometimes	172 (49.6)	142 (47.6)	
Never	155 (44.7)	140 (47.0)	
Missing data	3 (0.9)	1 (0.4)	
*Is the method easy to be used?*			
Yes	295 (85.0)	256 (85.9)	0.326
No	13 (3.7)	5 (1.7)	
Not sure	25 (7.2)	20 (6.7)	
Missing data	14 (4.1)	17 (5.7)	
*Satisfied with the method?*			
Yes	227 (65.4)	224 (75.2)	0.012
No	50 (14.4)	39 (13.1)	
Not sure	56 (16.1)	24 (8.0)	
Missing data	14 (4.1)	11 (3.7)	

Abbreviations: LNG-IUS, levonorgestrel-releasing intrauterine system; TCu380A IUD, T-shaped copper intrauterine device.

Data presented as *n* (%). *Pearson χ^2^ test.

When performing a multiple regression, we have observed that women who had ≤ 2 children were 4 times at risk for sexual dysfunction, those with < 8 years of schooling were at 1.7 time risk, and those classified as C, D or E in the household income classification were 1.6 time at risk for sexual dysfunction (Table 4).

**Table 4:** Multiple regression for sexual dysfunction

Selected variables	Categories	*p-*value	OR	95% CI
Group (covariates)	LNG-IUS	0.779	1.00	0.71–1.57
	TCu380A IUD		1.06	
Vaginal parity	≥ 3	0.006	1.00	1.49–10.52
	0–2		3.96	
Schooling (years)	> 8	0.020	1.00	1.09–2.67
	≤ 8		1.70	
Household income classification	A, B	0.029	1.00	1.05–2.34
	C, D, E		1.57	

Abbreviation: CI, confidence interval; TCu380A IUD, T-shaped copper intrauterine device; LNG-IUS, levonorgestrel-releasing intrauterine system; OR, odds ratio.

*Stepwise* criteria for variables selection.

Since this was the data analysis of part of a larger study, we have analyzed the power of the sample size taking into consideration the proportion of sexual dysfunction and of QOL between the 2 IUCs with the significance of 5%. We have observed the power of 36.5% for sexual dysfunction, of 99.9% for the physical, environmental, and overall QOL domains, 13.8% for the psychological domain, and 6.3% for the social relationships' domain.

## Discussion

Among the Cu-IUD and the LNG-IUS users, 35.2% and 29.2% presented a FSFI score < 26.55, respectively, indicating a risk for sexual dysfunction; however, no differences were found when comparing both groups.

Enzlin et al.^12^ assessed the sexual function among Cu-IUD and LNG-IUS users with a different questionnaire, the Short Sexual Function Scale, and did not find differences between the two groups.

In a previous observational study, the authors analyzed if the 31 users of the LNG-IUS presented any difference in QOL and sexual function 12 months after the insertion of the IUC, and no effect on those variables were found.^13^ Koseoglu et al^2^ compared the sexual function among a group of 92 TCu380A IUD-users and a group of 83 women who did not use contraceptives. The FSFI questionnaire was also used in the study, and the score among the TCu380A IUD users was lower when compared with the control group; however, the difference was not significant. When we compared the two IUC groups, we have also found a lower FSFI score in the TCu380A IUD group when compared with the LNG-IUS group; however, this difference was also not significant. More women in Group 1 (32 [9.2%]) than in Group 2 (12 [4.0%]) had 3 to 4 vaginal deliveries; however, this may not be associated with a worse sexual function, as has been shown in a previous study in which no negative effect on the sexuality of women after vaginal delivery was found.^14^

A prospective study evaluated 158 LNG-IUS users, in which the participants answered the FSFI questionnaire at the beginning of the survey and after 1 year of use.^8^ There were no significant differences between the two moments of response to the questionnaire. Although the FSFI is considered the gold standard to determine female sexual function,^2^ this questionnaire may miss some important aspects such as psychological and subjective factors or sexual aspects of bleeding and cramping,^15^ and may leave behind some important aspects regarding female sexual function.

In addition, a prospective observational study assessed the sexual function and the QOL using the WHOQOL-BREF questionnaire among IUC-users either at the beginning of use or after 3 to 4 months after its placement.^3^ No difference was observed in the general aspects of life and in sexual function, which could be interpreted that the contraceptive methods did not affect these aspects in the lives of the participants.

When analyzing the mean scores of the WHOQOL-BREF questionnaire, the TCu380A IUD group had a significant lower score in the physical health, environmental, and overall QOL domains in comparison with the LNG-IUS group. Li et al have also used the WHOQOL-BREF questionnaire to assess the QOL among first-time users of various contraceptive methods. Regarding the IUC users, no impact on the QOL was found.^3^

In the present study, we did not assess information about menstrual patterns; however, considering that the TCu380A IUD could increase the menstrual flow, we can speculate that this was one of the reasons for the lower score in some of the domains of QOL among the women in Group 1; meanwhile, the opposite happens regarding the use of the LNG-IUS, which may explain the better score in the WHOQOL-BREF questionnaire in Group 2. There is evidence that users of the LNG-IUS were satisfied with the non-contraceptive benefits, especially when it improved the menstrual patterns among women experiencing heavy or prolonged bleeding.^8^ This can also explain our finding concerning the satisfaction with the method in use when the participants answered to the questionnaire formulated for the present study. More women in Group 1 were not satisfied or were not sure about their satisfaction with the contraceptive method used when compared with Group 2.

Additionally, more women in Group 1 were non-white, had < 8 years of schooling, and had a lower household income. These sociodemographic characteristics could influence the lower QOL domains. The fact that more women using the LNG-IUS had > 8 years of schooling could indicate that they were more informed and could reach a health care professional (HCP) to help them if something was wrong in these aspects of their lives.

The main strength of the present study was the comparison of both IUCs. To the best of our knowledge, there are no studies that have evaluated both sexual function and QOL among TCu380A IUD and LNG-IUS (20 gµ/day initial release) users. Also, the questionnaires were self-responded, which excluded the possibility of courtesy bias or of possible external influence on the participants. The main limitations were that only one questionnaire about sexuality and QOL was used; lack of exclusion of women with personal history of sexual dysfunction before the placement of the method; not presenting the time of IUC use in each group; and not establishing a minimum time of IUC use for the inclusion in the study.

## Conclusion

Sexual function did not differ between TCu380A IUD and LNG-IUS users; however, TCu380A IUD users scored less in some QOL domains. It is not possible to ensure that those differences were related to the method in use. Maybe with more specific evaluation tools, it would be possible to assess more information related to the sexual function and to the QOL of the participants relating specifically with the contraceptive in use. The findings of the present study could be useful for HCPs at the time of counseling to users and to potential users of IUCs.
